# Feasibility of Augmenting Ankle Exoskeleton Walking Performance With Step Length Biofeedback in Individuals With Cerebral Palsy

**DOI:** 10.1109/TNSRE.2021.3055796

**Published:** 2021-03-02

**Authors:** Ying Fang, Zachary F. Lerner

**Affiliations:** Department of Mechanical Engineering, Northern Arizona University, Flagstaff, AZ 86011, USA.; Department of Mechanical Engineering, Northern Arizona University, Flagstaff, AZ 86011, USA; Department of Orthopedics, The University of Arizona College of Medicine-Phoenix, Phoenix, AZ 85004, USA

**Keywords:** Biofeedback, exoskeleton, rehabilitation, walking ability

## Abstract

Most people with cerebral palsy (CP) suffer from impaired walking ability and pathological gait patterns. Seeking to improve the effectiveness of gait training in this patient population, this study developed and assessed the feasibility of a real-time biofeedback mechanism to augment untethered ankle exoskeleton-assisted walking performance in individuals with CP. We selected step length as a clinically-relevant gait performance target and utilized a visual interface with live performance scores. An adaptive ankle exoskeleton control algorithm provided assistance proportional to the real-time ankle moment. We assessed lower-extremity gait mechanics and muscle activity in seven ambulatory individuals with CP as they walked with adaptive ankle assistance alone and with ankle assistance plus step-length biofeedback. We achieved our technical validation goal by demonstrating a strong correlation between estimated step length and real step length (R = 0.771, p *<* 0.001). We achieved our clinical feasibility goal by demonstrating that biofeedback-plus-assistance resulted in a 14% increase in step length relative to baseline (p ≤ 0.05), while no difference in step length was observed for assistance alone. Additionally, we observed near immediate improvements in lower-extremity posture, moments, and positive power relative to baseline for biofeedback-plus-assistance (p *<* 0.05), with none, or more-limited improvements observed for assistance alone. Our findings suggest that providing real-time biofeedback and using step length as the target can be effective for increasing the rate at which individuals with CP improve their gait mechanics when walking with wearable ankle assistance.

## I. Introduction

INDIVIDUALS with cerebral palsy (CP) experience movement and posture impairment that negatively affects their mobility and physical activity levels [1], [2]. They often exhibit pathological gait patterns that can involve excessive knee and hip flexion during stance and reduced ankle plantarflexion prior to push-off. These gait patterns are associated with slow walking speed, reduced step length and increased energy cost of transport [3]–[5]. Many children with CP experience a steady decline in mobility across their lifespan [6].

Functional gait training has received significant attention from clinical researchers aiming to develop effective interventions for children with CP [7], [8]. There is a growing consensus that functional gait training interventions must reinforce improved movement patterns resulting from enhanced engagement as opposed to passive repositioning of limbs through the use of robot-assisted gait trainers [9]. As such, gait training with biofeedback is a technique that has been used with some success to incentivize individuals to volitionally change their walking behavior. Improvement in joint angles [10], [11], spatiotemporal characteristics [12] and muscle activity [13] are possible when these parameters are used as direct biofeedback targets in gait training for people with CP. Recently, step length has been found to be an intuitive and effective feedback modality that also enhanced ankle power generation in CP [14].

Wearable assistive devices also have potential to reinforce favorable movement patterns and increase training dose by making walking easier for people with neuromuscular conditions [15], [16]. We previously demonstrated that an untethered ankle exoskeleton can increase ankle plantarflexion power, improve walking posture, and reduce the metabolic cost of treadmill walking in individuals with CP [16]. However, our research has demonstrated that repeated practice is required to observe improved gait biomechanics during walking with assistance [17] and, when assistance is tuned to reduce the metabolic cost of transport, individuals with CP walk with reduced stance phase ankle plantar-flexor muscle activity [18]. A logical concern is that users may become reliant on wearable assistance over time, leading to muscle atrophy and reduced function.

Incorporating biofeedback with body-weight-supported or tethered robot-assisted (i.e., Lokomat) gait training has been found to facilitate anticipated favorable rehabilitation benefits in people with stroke [19], [20], spinal cord injury [21], and in children with CP [22]. However, we are not aware of any study that has integrated biofeedback with wearable (i.e., untethered) powered assistive devices to promote training outcomes. Integrating biofeedback techniques with wearable interventions that can deliver both daily mobility assistance and functional gait training holds potential to facilitate long-term functional gains in clinical and home settings.

Working towards our overarching goal of increasing the effectiveness of gait training with wearable assistance, the purpose of this study was to develop and validate the feasibility of a real-time biofeedback mechanism to augment untethered ankle exoskeleton-assisted walking performance in individuals with CP. We hypothesized that combining step-length biofeedback with adaptive plantar-flexor assistance would encourage users with CP to walk with longer strides through increased plantar-flexor muscle activity and greater positive joint power across the lower-extremity when compared to their baseline gait pattern and walking with ankle assistance alone.

## II. Methods and Procedures

### A. Overview

We sought to develop a practical, effective biofeedback system to augment walking with ankle exoskeleton assistance. The primary design criteria for our exoskeleton biofeedback system included (1) a clinically-relevant gait performance target, (2) a simple, intuitive user interface suitable for children, (3) the inclusion of simple gamification techniques to incentivize participation, and (4) ankle exoskeleton assistance that accommodated instantaneous changes in gait mechanics. The system we implemented utilized real-time step length information, the user’s instantaneous performance relative to a personalized goal, a visual interface with live performance score, and an adaptive ankle exoskeleton control algorithm that provided assistance proportional to the real-time ankle moment.

Step-length was selected as the gait performance target because it (1) is a common clinical goal for individuals with CP [5], (2) encourages ankle push-off and lower-extremity extension [23], (3) is relatively simple to implement and intuitive for users of all ages. A previous study on children with CP tried multiple biofeedback targets and concluded that step length was the most intuitive among all options, and which resulted in increased ankle power in their cohort [14].

### B. Ankle Exoskeleton

**Fig. 1.**
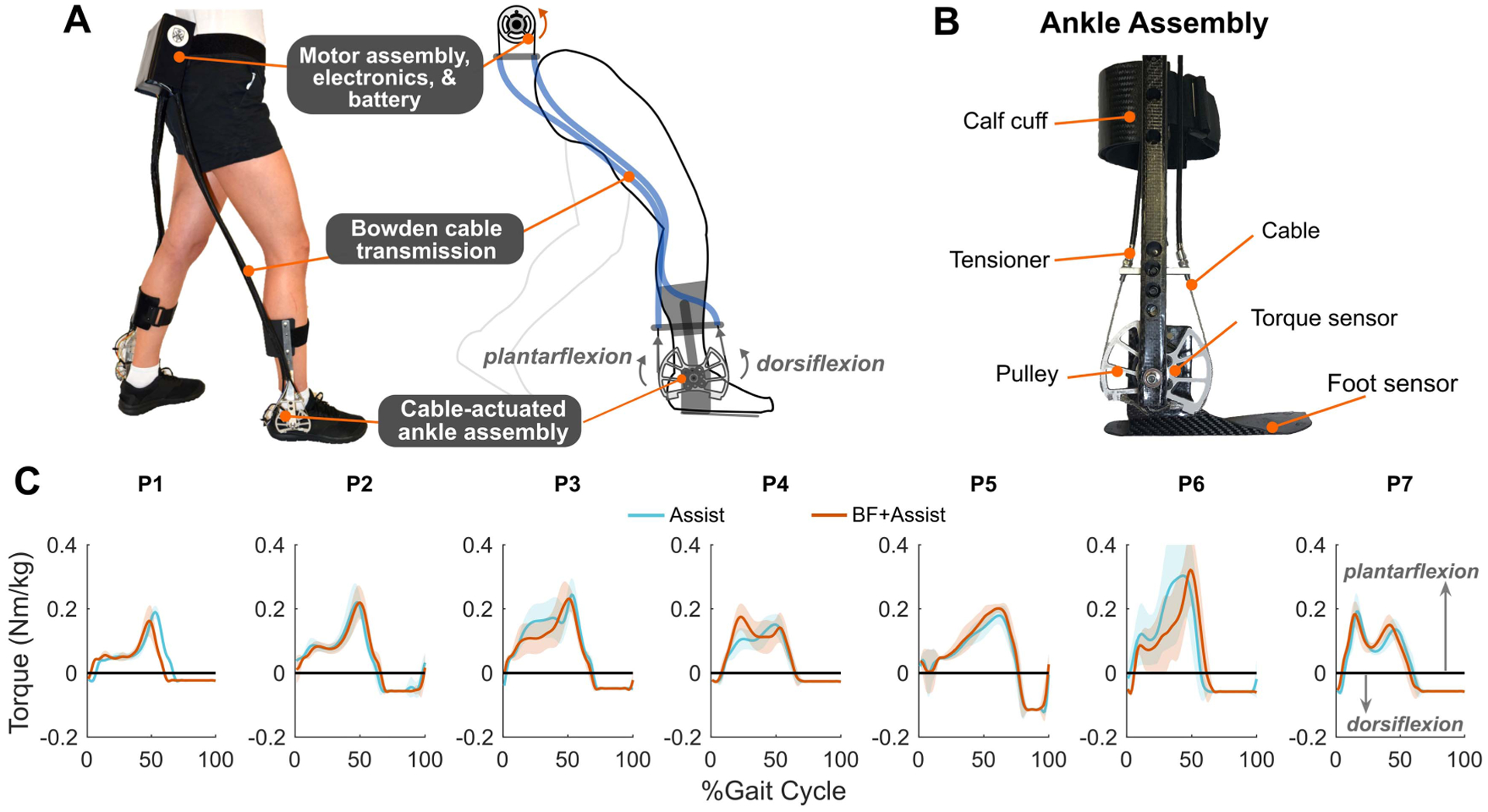
A) Mechanical design of the ankle exoskeleton wore by the users. B) Components of the ankle assembly of the exoskeleton. C) The amount of plantarflexion and dorsiflexion torque received by each participant.

An untethered ankle exoskeleton, customized for each participant (mass: 1.85 kg for the small size and 2.20 kg for the large size), was used to provide ankle assistance and collect information for real-time biofeedback. Detailed information about the adaptive controller [24] and electromechanical [25] design of the device has been provided previously. The fundamental premise of the system is to improve ambulatory function by augmenting plantar-flexor deficits in CP using a control strategy that automatically accounts for stride-to-stride variation to achieve seamless synchronization between the device and user. In brief, a waist-mounted motor and control assembly remotely actuated carbon fiber ankle assemblies via a Bowden cable transmission (Fig. 1A, B). A finitestate-machine was used to identify each stance and swing phase using embedded foot sensors (force sensitive resistors; FSR). We estimated the real-time biological ankle moment using a previously-validated control strategy based on the FSRs [24]. During stance phase, the exoskeleton provided adaptive plantar-flexor torque proportional to the real-time estimate of the biological ankle moment, reaching a peak of 0.22 ± 0.06 Nm/kg, on average. During swing phase, 0.02 to 0.11 Nm/kg of dorsiflexion assistance was provided based on user preference and visual assessment of toe clearance (Fig. 1C).

### C. Real-Time Biofeedback

**Fig. 2.**
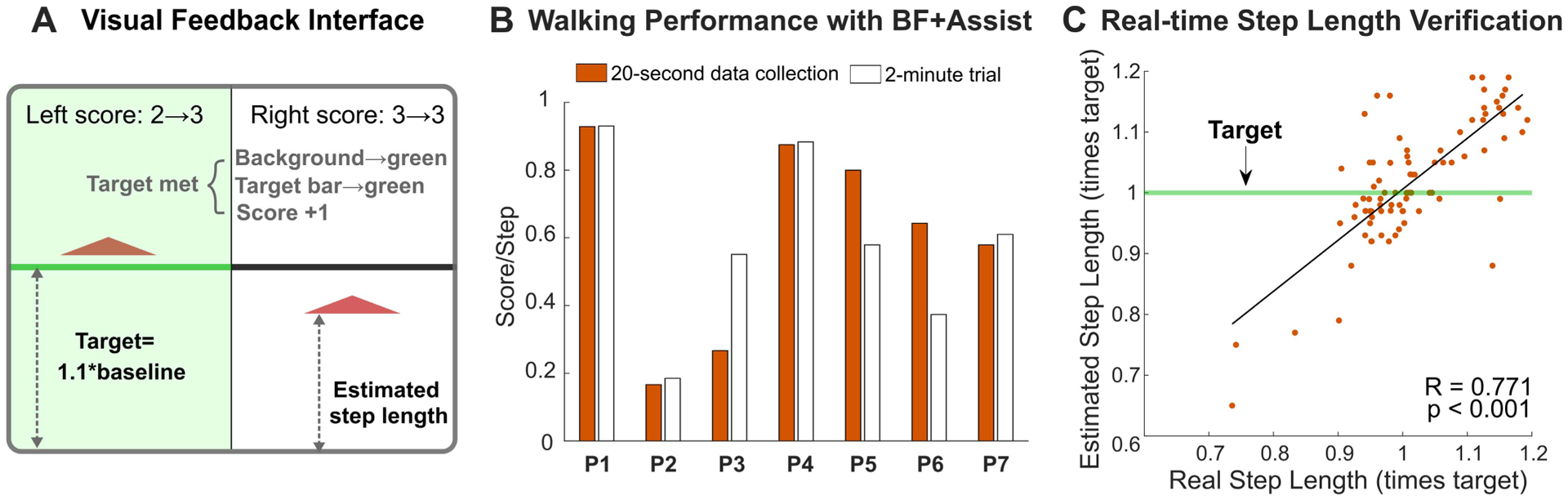
A) Depiction of the biofeedback visual interface. The vertical position of the two triangles indicated the average length of the most recent two steps for the left and right legs. The horizontal bar indicated the target step length. If a user reached the target, the background and target bar turned green and one point was added to the score. B) Cumulative scores achieved by each participant during walking with biofeedback-plus-assistance (BF+Assist) throughout the 2-minute trial and within the 20-second data collection period. C) Estimated step length by the foot sensor and experimentally measured step length from the motion capture system.

We developed a visual biofeedback system to provide real-time step length information to each user. The length of each step was approximated as follows:

(1) $$\text{step lengt}h_{n}=\text{walking speed}\times (t_{n}-t_{n-1})$$

where t_n_ was the time at foot contact for the n^th^ step. Time at foot contact, recorded bilaterally using the exoskeleton’s foot sensors, was transmitted via Bluetooth to a laptop computer running a MATLAB graphical user interface. The laptop was connected to a large (1.42 m by 0.80 m) display that was placed 3 m in front of the treadmill (Supplemental Fig. 3). On the visual interface, symbols moved up and down with their vertical position representing the relative length of the left and right step. A horizontal bar indicated the target step length, which was set 10% greater than during baseline walking. The display showed the two-step moving average, and was updated every two strides (Fig. 2A). The user’s score was reported at the top of the display. Points were earned if the average length of the last two steps exceeded the target (Fig. 2B).

### D. Participants and Study Design

Seven individuals with CP between 6 and 31 years of age participated in the study (Table I). Inclusion criteria included diagnoses of CP; the ability for participants to walk on a treadmill with or without a walking aid for at least 6 minutes; gross motor function classification system (GMFCS) level I, II, or III; at least 20° of passive ankle plantarflexion range of motion; no knee extension or ankle dorsiflexion contractures greater than 15°; no orthopedic surgery completed in the prior 6-month; and the absence of any medical condition other than CP that would affect safe participation. The study was approved by the Institutional Review Board of Northern Arizona University (NAU) under protocol #986744 on 5/13/2019 as a part of NCT04119063. Participants over 18 years old read and signed an informed consent document. For each minor, we obtained assent and informed written consent from a parent.

Following the consent process, we determined each participant’s baseline preferred treadmill walking speed and step length and explained the concept of biofeedback and the visual interface. Next, participants completed the following three walking conditions at the fixed, pre-determined baseline-preferred speed in random order: baseline – walking wearing shoes and orthoses if they were prescribed by a physician; assistance alone – walking with the exoskeleton as it provided bilateral plantar- and dorsi-flexor assistance; biofeedback-plus-assistance – walking with biofeedback wearing the exoskeleton as it provided both plantar- and dorsi-flexor assistance. Participants walked for two minutes under each condition and for one minute between conditions wearing the exoskeleton with no biofeedback or assistance as a washout. Breaks were provided as needed. This validation study was completed on a treadmill to isolate the effects of biofeedback and exoskeleton assistance when walking at the same speed.

We collected motion, force, and muscle activity data for the final 20 seconds of each condition. A ten-camera motion capture system (120 Hz; Vicon) with a custom marker set was used to record kinematics data [16], [26]. We placed markers bilaterally on the mid-toe, heel, 5th metatarsal, medial and lateral malleolus, medial and lateral epicondyle, anterior and posterior superior iliac spine, and acromion process, and on the sternum and 7th cervical vertebra. Clusters with four markers were placed on the shank and thigh of each leg. We collected ground reaction force using an instrumented treadmill (980 Hz; Bertec). Muscle activity was recorded from the soleus and vastus lateralis using a wireless electromyography (EMG) system (1926Hz; Delsys). Experimental data were recorded simultaneously in the Vicon system and its Nexus software and synchronized with exoskeleton data (100 Hz) using a trigger signal.

### E. Data Analysis

We identified gait events (heel strike and toe-off) in Vicon Nexus and calculated the length of each step based on the coordinates of left and right heel markers at heel strike. We used OpenSim 3.3 [27] to derive joint kinematics and kinetics. We first scaled a generic musculoskeletal model [28] for each participant, and then computed the joint angles and moments across the lower-extremity using the inverse kinematics and inverse dynamics analyses. We derived the support moment by summing the time-series data of ankle, knee, and hip moments [29], [30]. Joint power (W) was calculated as the product of the joint moment (Nm) and the respective joint angular velocity (rad/s). Stance-phase average positive power was calculated by integrating the positive area of the joint power curve and dividing by stance time.

EMG data were band-pass filtered between 15 and 380 Hz, rectified, and low-pass filtered with a 7 Hz cutoff to generate the linear envelope [31]. We normalized the filtered EMG signal for each muscle by the peak value from walking with the device unpowered (zero torque). EMG data were then segmented into gait cycles. The area under the EMG curve (integrated EMG, iEMG) was summed for the stance phase and divided by the period as an indication of muscle work [32].

Primary outcomes included step length; peak ankle plantarflexion, knee extension, and hip extension during stance phase; peak stance phase ankle, knee, hip, and support moments; average positive individual and summed ankle, knee, and hip power during stance; and stance phase soleus and vastus lateralis iEMG. Total (combined muscle and exoskeleton contributions) and biological (muscle contribution) joint moment and power at the ankle were computed for exoskeleton-assisted trials [16]. The torque (measured using torque sensors) and power (product of measured torque and angular velocity) from the device were subtracted from the joint moment and power, respectively, from inverse dynamics, as in [33] to derive biological moment and power. Kinetic outcomes were normalized by body mass. All outcomes were averaged within limbs and across all gait cycles of the 20-second data collection.

### F. Statistical Analysis

To evaluate the accuracy of the biofeedback mechanism, we calculated the root mean square error (RMSE) and Pearson’s correlation coefficient (R) between real-time estimate and motion-capture-measured step length of all participants; coefficients of 0.7 – 0.9 were considered strong correlations [34]. We used paired two-tailed t-tests to compare outcomes between the biofeedback-plus-assistance and assistance alone conditions, between biofeedback-plus-assistance and baseline, and between assistance alone and baseline. We calculated Pearson’s correlation coefficient (R) between baseline peak biological ankle moment and change in peak biological ankle moment during biofeedback-plus-assistance relative to baseline. Kolmogorov-Smirnov tests were used to check the normality of the outcomes in each comparison (Supplemental Table I). For parameters that were not normally distributed, we used Wilcoxon signed-rank tests to evaluate statistical significance. A fixed significance level of *α* ≤ 0.05 was used for this feasibility study.

## III. Results

### A. Biofeedback Efficacy

There was a strong correlation between the step length estimated from the real-time biofeedback system and the step length computed from motion capture (R = 0.771, p *<* 0.001); the RMSE between estimated and measured values was 6.3 ± 3.3% across the seven participants (Fig. 2C).

### B. Effect of Biofeedback During Walking With Assistance

**Fig. 3.**
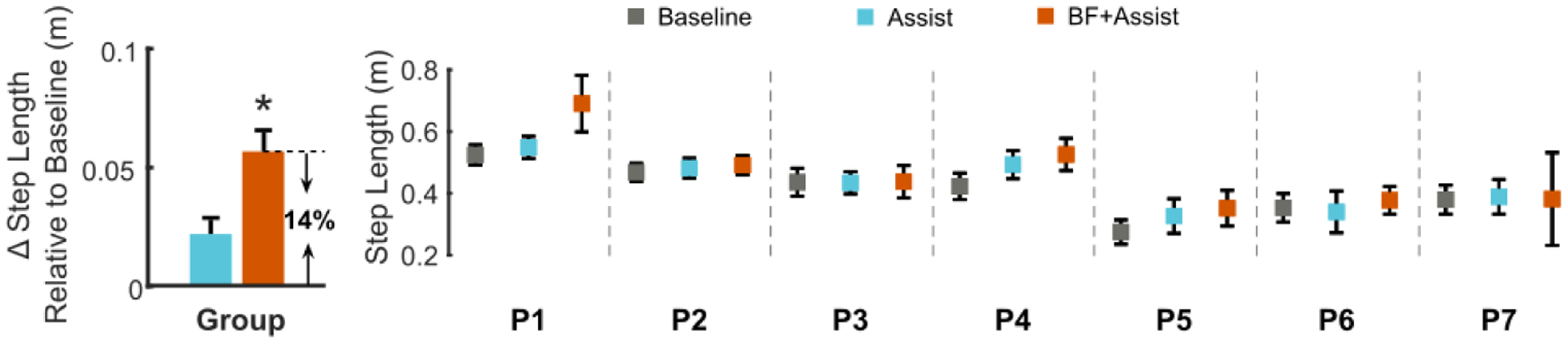
(Left) Group-level changes in step length relative to baseline during walking with assistance alone (Assist) and biofeedback-plus-assistance (BF+Assist) conditions (mean + standard error). * Indicates statistically significant difference from the baseline. (Right) Plots of step length (mean ± standard deviation) for each participant across conditions.

Participants increased their step length by 13.8 ± 11.8%, averaged across both limbs, when they walked with biofeedback-plus-assistance compared to baseline (p = 0.05); step length was similar between walking with biofeedback-plus-assistance and assistance alone (p = 0.114) and between assistance alone and baseline (p = 0.096, Fig. 3).

**Fig. 4.**
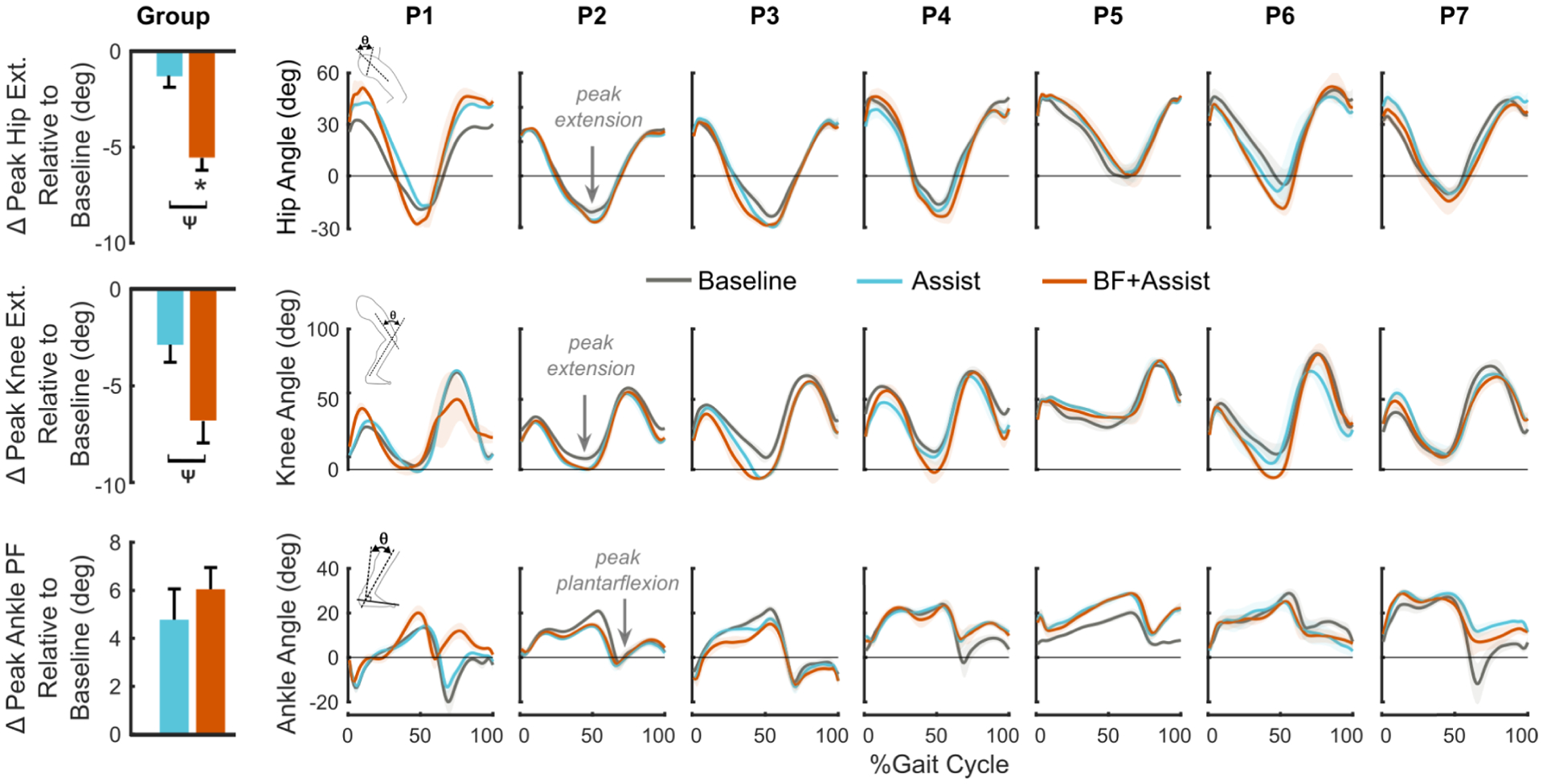
(Left) Group-level changes in stance-phase peak hip extension (top), peak knee extension (middle), and peak ankle plantarflexion (bottom) across both limbs for walking with only ankle assistance (Assist) and with biofeedback-plus-assistance (BF Assist) relative to walking wearing shoes (Baseline). * Indicates statistically significant difference from the baseline, and 9 indicates statistical significance between Assist and BF+Assist. Error bars indicate standard error. (Right) Plots of hip, knee, and ankle angle for each participant’s right leg across conditions. Shading depicts mean ± standard deviation.

Walking with biofeedback-plus-assistance contributed to a more extended lower-extremity posture. Late-stance hip extension increased by 4.2 ± 5.1° compared to walking with assistance alone (p = 0.028) and by 5.5 ± 4.7° compared to baseline (p = 0.018, Fig. 4). Additionally, walking with biofeedback-plus-assistance increased stance phase knee extension by 3.9 ± 3.6° compared to assistance alone (p = 0.028). Late-stance peak ankle plantarflexion angles were similar across conditions (Fig. 4).

**Fig. 5.**
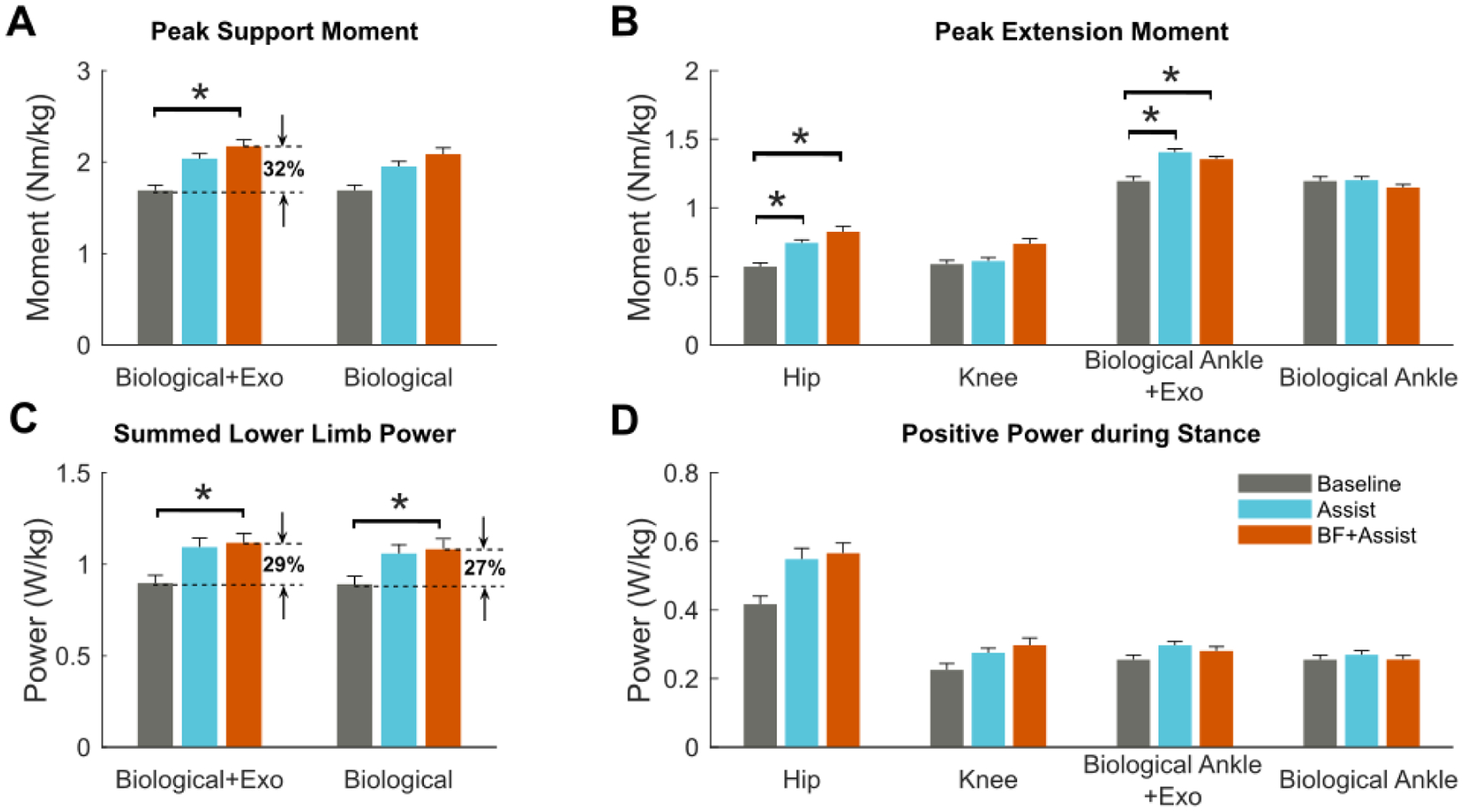
A) Peak lower extremity total support moment (hip, knee, biological ankle, and exoskeleton) and peak lower extremity biological support moment (hip, and knee, biological ankle); B) peak stance phase hip extension moment, knee extension moment, ankle (biological and exoskeleton) plantarflexion moment, and biological ankle plantarflexion moment; C) summed positive power for the lower extremity (hip, knee, biological ankle, and exoskeleton) and biological lower extremity (hip, and knee, biological ankle); and D) positive joint power for the hip, knee, ankle (biological and exoskeleton), and biological ankle during the stance phase, across both limbs for baseline walking with shoes (Baseline), with only ankle assistance (Assist), and with Biofeedback-plus-Assistance (BF+Assist). * Indicates statistical significance. Error bars indicate standard error.

Walking with biofeedback-plus-assistance resulted in increased lower-extremity extensor moments and powers. Compared to baseline, stance phase peak hip extension and ankle plantarflexion moments increased by 50.6 ± 42.9% (p = 0.041) and 15.7 ± 17.7% (p = 0.03), respectively. Walking with biofeedback-plus-assistance increased the peak support moment summed across the lower-extremity by 31.7 ± 35.1% (p = 0.039) compared to baseline; similarly, total (biological + exo) lower-extremity positive power increased 29.1 ± 25.3% (p = 0.03) and summed biological positive power increased 26.5 ± 25.3% (p = 0.045). Walking with ankle assistance alone did not result in an increased peak support moment or summered lower-extremity power, but did increase peak hip and ankle moments by 36.5 ± 32.9% (p = 0.036) and 20.0 ± 19.0% (p = 0.023), respectively. There were no statistically significant differences between biofeedback-plus-assistance and assistance alone conditions with respect to joint moments or powers (Fig. 5, Supplemental Fig. 1 & 2). We observed a negative relationship between baseline biological ankle moment and relative changes in biological ankle moment measured when walking with biofeedback-plus-assistance (R = 0.758, p = 0.048, Fig. 7).

**Fig. 6.**
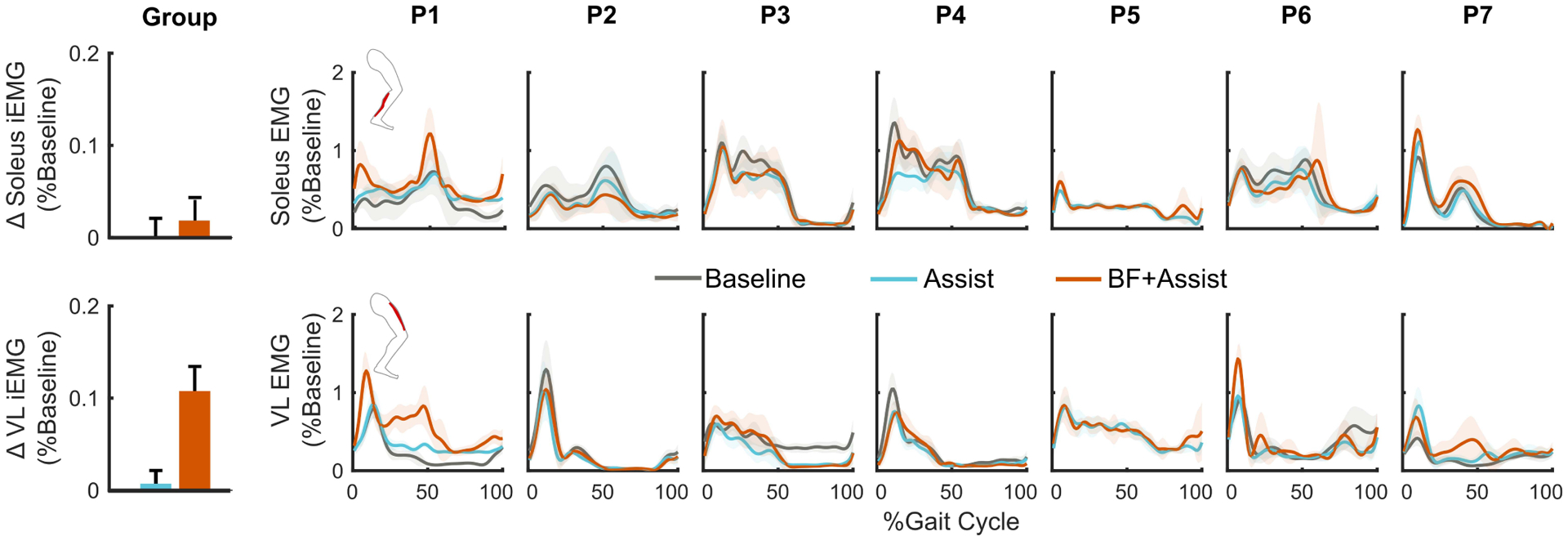
(Left) Group-level changes in stance phase integrated muscle activity (iEMG) for the soleus (top) and vastus lateralis (VL; bottom) across both limbs when walking with only ankle assistance (Assist) and biofeedback-plus-assistance (BF+Assist) relative to baseline walking with shoes (Baseline). Error bars indicate standard error. (Right) Plots of normalized soleus activity (top) and VL activity (bottom) of each participant’s right leg across conditions. Shading depicts mean ± standard deviation.

There were no significant differences in soleus or vastus lateralis iEMG across conditions (Fig. 6).

## IV. Discussion

The overarching goal of this study was to develop and validate a real-time biofeedback mechanism to augment untethered ankle exoskeleton-assisted walking performance in individuals with CP. As the first study combining untethered exoskeleton assistance with real-time biofeedback in individuals with CP, we achieved our goal of establishing the feasibility of this gait training intervention and improving clinically-relevant outcomes. Fulfilling our validation goal, there was a strong correlation between the step length estimated from the real-time biofeedback system and the step length computed from motion capture (R = 0.771), with low average RMSE (6.3%). We partially accept our hypotheses. Combining step length biofeedback with ankle assistance elicited longer steps, a more extended lower-extremity, and increased joint moments and powers. However, there were no statistically significant changes in muscle activity across conditions.

Our participants, with ages ranging from 6 to 31 years old and GMFCS levels between I to III, were able to understand and safely walk with the biofeedback system and ankle exoskeleton. We did not find a significant relationship between either age (R^2^ = 0.494, p = 0.078) GMFCS level (R^2^ = 0.365, or p = 0.151) to changes in step length. Anecdotally, we observed that enthusiasm for the biofeedback “game” positively influenced performance. For example, children and young adults who showed an earnest interest in the biofeedback feature had higher scores and took longer steps, while the oldest participant (P2, 31 years old) was visibly disinterested and earned the lowest score and had minimal changes in gait outcomes (Fig. 2B). We purposefully kept the step-length target constant in this feasibility analysis to mitigate the effects of personalized or variable targets as a confounding factor. Future exploration should investigate the role of personalized targets on user engagement and performance. Similar to previous studies [11], [14], two-minute acclimation time under biofeedback conditions for CP is reasonable as it provides sufficient time to get used to the feature and prevents fatigue. Most participants maintained or improved their performance throughout the 2-minute trial (Fig. 2B).

Combining biofeedback with assistance was effective in eliciting near immediate improvements in clinically-relevant measures of gait mechanics. In agreement with prior studies that have reported improvements of 9 – 13% for step length biofeedback in individuals with CP [12], [14], our cohort walked with 13.8 % longer steps with biofeedback-plus-assistance. Compared to walking with assistance alone, all but one participant (P7) achieved longer steps during biofeedback-plus-assistance (Fig. 3). Step length as the feedback target encourages simultaneous increases in step length and lower-extremity extension, while also giving users the autonomy to explore their own strategy to achieve that goal [14]. We found that our participants achieved longer steps during biofeedback-plus-assistance through increased hip (5.5°) and knee (3.9°) extension relative to baseline. Importantly, walking with biofeedback-plus-assistance increased the peak support moment summed across the lower-extremity (31.7%), and summed total (biological + exo, 29.1%) and biological (26.5%) lower-extremity positive power, which were improvements not observed for assistance alone. Analysis of the biofeedback-only condition suggests that step-length biofeedback encouraged increased hip extension regardless of ankle assistance (Supplemental Table II).

Part of our motivation for implementing biofeedback was to ensure user engagement during walking and prevent users’ reliance on the device. Our previous study observed reduced plantar-flexor muscle activity after over two hours of walking with the device [16]. Statistically significant changes in muscle activity across the lower-extremity were not observed, likely due, in part, to the inherent variability of this measure in this heterogeneous patient population. Still, the improvement in gait mechanics without an observed reduction in muscle activity during biofeedback-plus-assistance is an encouraging finding that suggests our intervention warrants additional investigation for functional gait training.

**Fig. 7.**
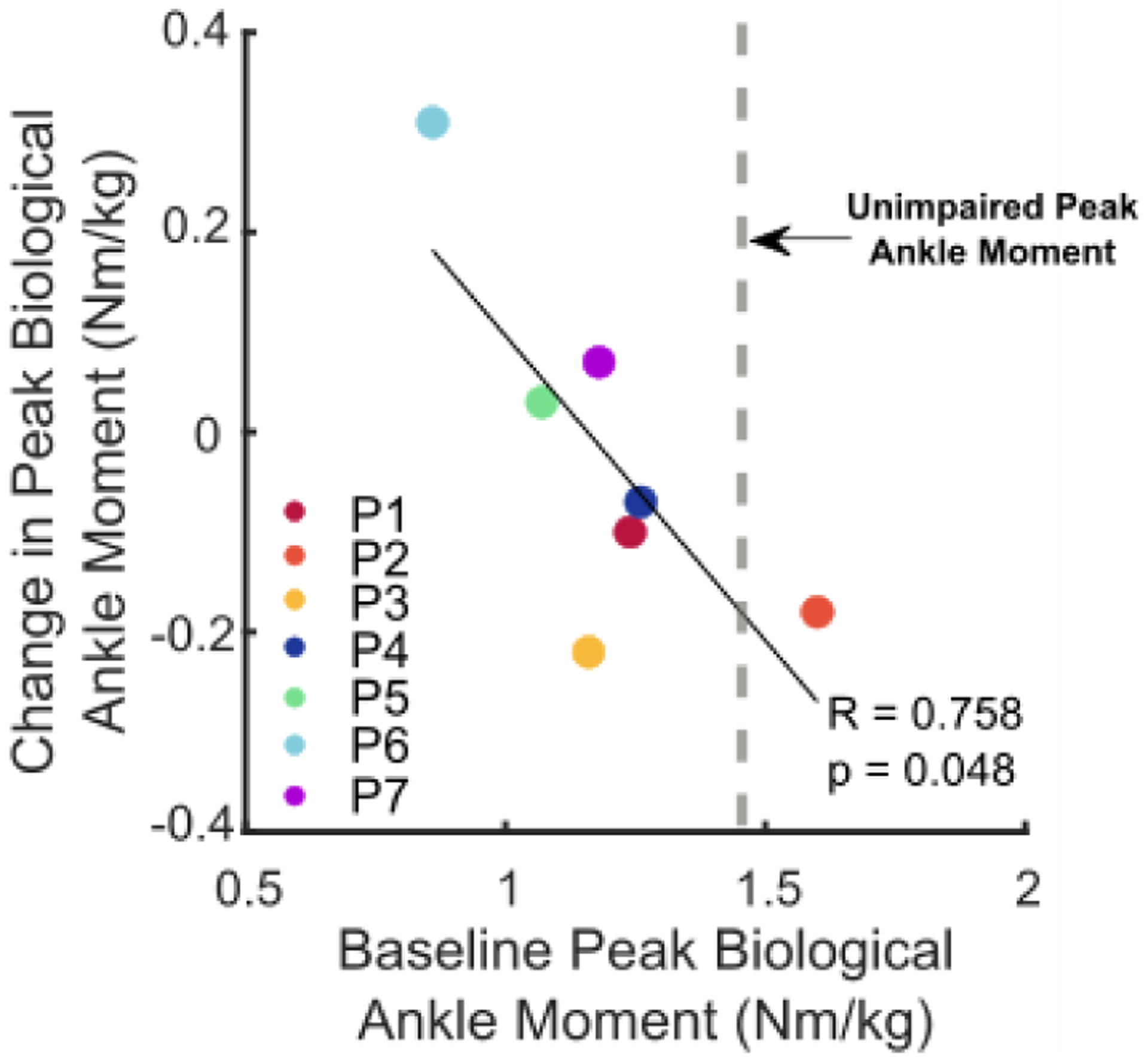
Correlation between baseline peak biological ankle moment and changes in peak biological ankle moment of biofeedback-plus-assistance relative to baseline.

Our results indicate that biofeedback could be an effective tool to augment clinically-relevant outcomes during walking with wearable assistance. In contrast with Booth *et al.* [14], we did not find an anticipated improvement in biological ankle power or moment compared to baseline or assistance alone. However, a comparison to walking with only biofeedback (Supplemental Table II) revealed that biofeedback-plus-assistance improved positive ankle power generation (both total and biological), which reinforces the anticipated benefits of this combined gait training modality. The lack of increased ankle power for biofeedback-plus-assistance vs. baseline in our study may be because the exoskeleton’s plantar-flexor torque immediately enhanced local function by increasing the total (exoskeleton + biological) ankle moment or power (Fig. 5, Supplemental Fig. 1). This theory is further supported by our finding that participants with biological plantar-flexor moments during baseline that were most similar to the typical range from unimpaired individuals had less improvement in this parameter when walking with biofeedback-plus-assistance (Fig. 7). This suggests an upper limit for which an individual may increase their total plantar-flexor moment for achieving longer steps.

A limitation of this validation study was the relatively small number of participants. However, the sample size (n = 7) was selected based on similar prior feasibility studies in the same population [15], [16], [36]. Our results suggest that step length biofeedback and ankle assistance was appropriate for a relatively broad range of individuals with CP; our cohort encompassed ages from 6 – 31 years old and GMFCS levels of I – III. We did not correct for multiple comparisons for this exploratory study. We did not test multiple trials for each condition. However, this design was consistent with previous biofeedback feasibility experiments in CP and older adults, where each condition lasted 1- or 2-minute and was tested only once [14], [37]. A necessary limitation was that the walking speed was kept constant for all conditions to isolate the biomechanical effects of biofeedback from changes induced by altered walking speed. We expect participants would have naturally walked faster when engaging with both steps length biofeedback and assistance. Increasing step length at a fixed speed on a treadmill would likely be a common implementation of this intervention in a rehabilitation setting because it provides a controlled, safe environment that allows users to concentrate on the feedback modality, focus on one aspect of their gait pattern, and maximize the number of high-quality steps. As user performance progresses with training, we expect assistance would decrease or the step-length target would increase as needed to maintain progress. Step-length biofeedback with ankle exoskeleton assistance could be used outside the laboratory. For treadmill walking, a therapist could specify the set speed into the feedback controller. For over-ground walking, a link-segment model and IMU-measured segment could be used to estimate step length and inform the feedback controller.

## V. Conclusion

In conclusion, our results suggest that providing real-time step-length biofeedback when walking with wearable ankle assistance may be an effective gait training tool in CP, particularly for individuals with moderate-to-severe plantar-flexor impairment. We observed near immediate improvements in lower-extremity posture, moments, and positive power relative to baseline for biofeedback-plus-assistance, with none, or more-limited improvements observed for assistance alone. Our findings support future research to investigate the longitudinal effects of gait training with combined biofeedback and ankle assistance.

## Supplementary Material

supp1-3055796

## Figures and Tables

**TABLE I:** Participant Characteristics

Participant	Age (Years)	Sex	Height (m)	Mass (kg)	GMFCS Level	Walking Speed (m/s)	Gait Type
P1	13	M	1.51	43.8	I	1.00	Mild ankle PF dysfunction and bilateral crouch
P2	31	M	1.70	53.3	II	1.05	Moderate ankle PF dysfunction and bilateral crouch
P3	9	M	1.37	31.1	I	0.75	Mild ankle PF dysfunction and bilateral crouch
P4	10	M	1.39	38.5	I	0.85	Mild ankle PF dysfunction and bilateral crouch
P5	23	F	1.47	43.8	III	0.50	Severe ankle PF dysfunction and bilateral crouch
P6	6	M	1.10	17.2	II	0.65	Moderate ankle PF dysfunction and bilateral crouch
P7	6	M	1.18	17.6	I	0.85	Mild ankle PF dysfunction and right leg crouch

GMFCS: Gross Motor Function Classification System, PF: plantarflexion
