# Supplementary material for: Feasibility of Augmenting Ankle Exoskeleton Walking Performance With Step Length Biofeedback in Individuals With Cerebral Palsy
Source: IEEE Trans Neural Syst Rehabil Eng. Author manuscript; Available in PMC 2021 Mar 17. (PMC7968126; doi:10.1109/TNSRE.2021.3055796)
Supplement: supp1-3055796 [file NIHMS1679355-supplement-supp1-3055796.pdf]

Supplemental Data

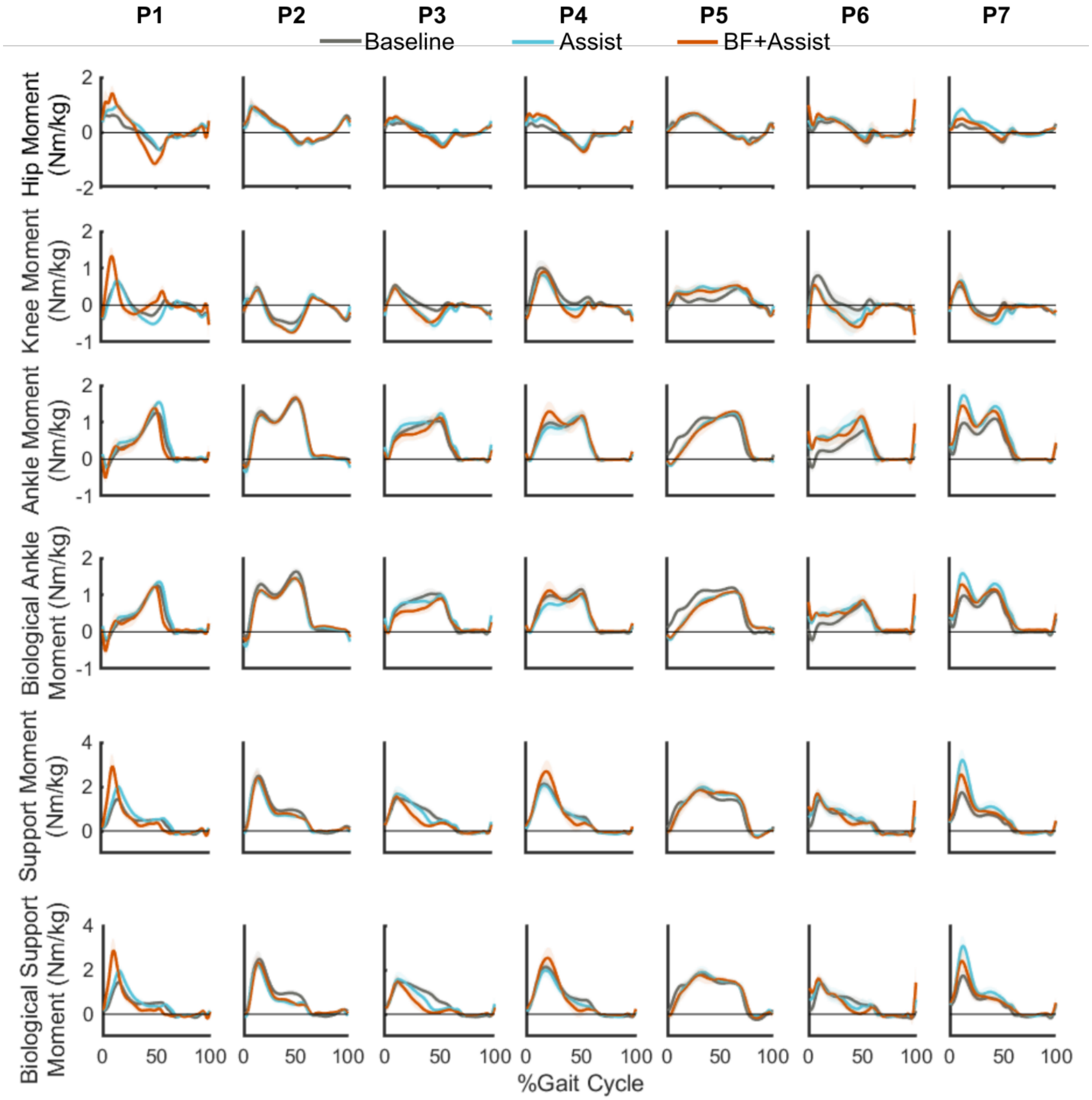

Supplemental Fig. 1. The curves show the hip, knee, ankle (exoskeleton + biological), biological ankle, support (hip + knee + biological ankle + exoskeleton) and biological support (hip + knee + biological ankle) moment for each participant's right leg for baseline walking with shoes (Baseline), with only ankle assistance (Assist), and with Biofeedback-plus Assistance (BF+Assist). Shading depicts mean ± standard deviation.

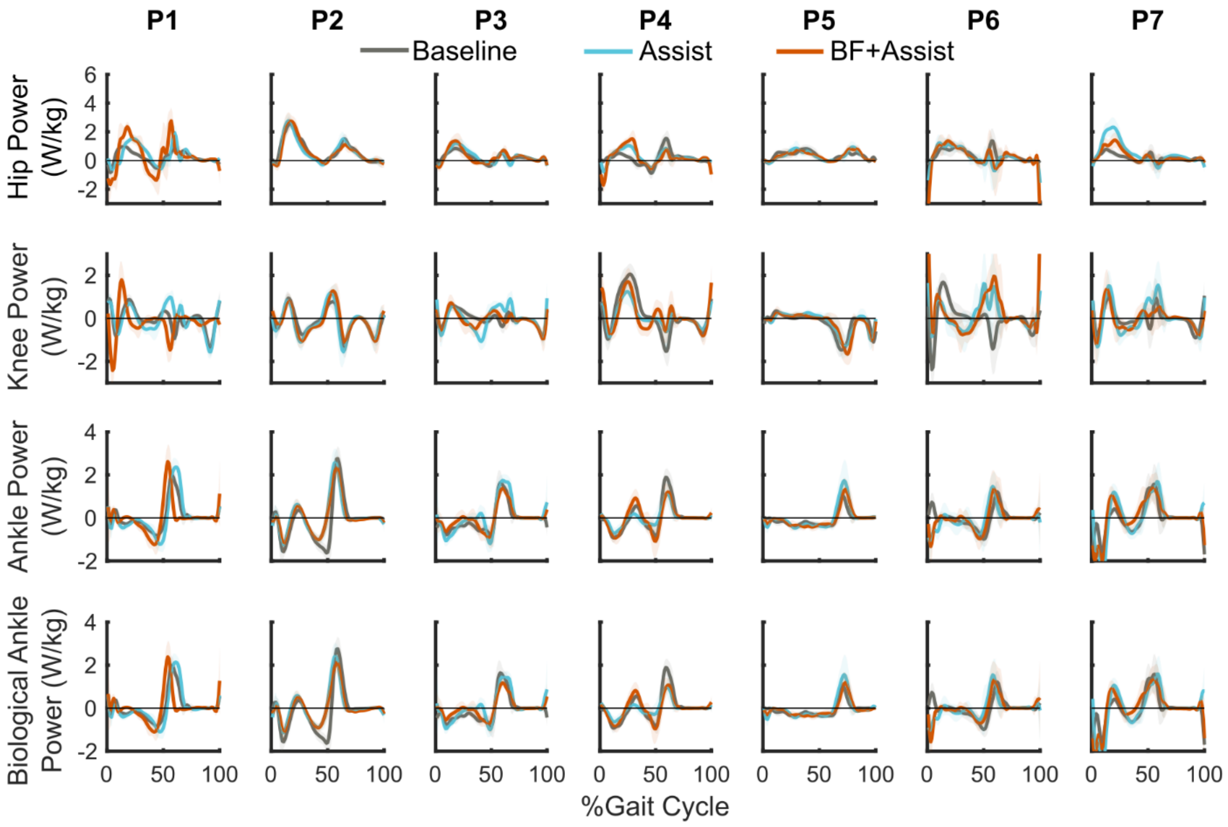

Supplemental Fig. 2. The curves show the hip, knee, ankle (exoskeleton + biological), biological ankle power for each participant's right leg for baseline walking with shoes (Baseline), with only ankle assistance (Assist), and with Biofeedback-plus Assistance (BF+Assist). Shading depicts mean ± standard deviation.

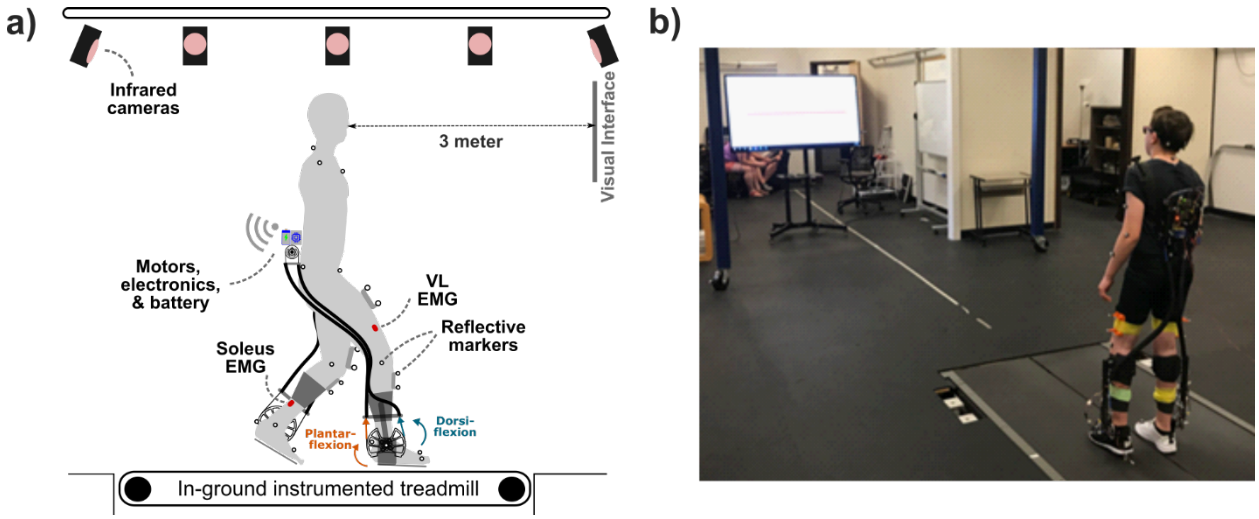

Supplemental Fig. 3. A) Depiction of the experimental set up and b) a photo showing a participant walked with biofeedback and ankle assistance. Please note that the biofeedback visual interface on the display does not show all of the visual information because the white background washed out most of the detail.

Supplemental Table 1. Normality following Kolmogorov-Smirnov tests. $P < 0.05$ indicates data that are not normally distributed.

Parameters	Baseline	Assistance	BF+Assistance
Step length	0.2	0.2	0.2
Peak Hip Extension Angle	0.2	0.2	***0.008***
Peak Knee Extension Angle	0.2	0.2	0.2
Peak Ankle Plantarflexion Angle	0.2	0.2	0.2
Peak Hip Extension Moment	0.091	0.166	0.2
Peak Knee Extension Moment	0.2	***0.02***	0.123
Peak Ankle Plantarflexion Moment	0.2	0.165	0.2
Peak Biological Ankle Plantarflexion Moment	0.2	***0.037***	0.2
Peak Support Moment	0.2	0.2	0.2
Peak Biological Support Moment	0.2	0.2	0.2
Average Positive Hip Power	0.2	0.2	0.2
Average Positive Knee Power	0.075	0.2	0.2
Average Positive Ankle Power	0.2	0.2	0.2
Average Positive Biological Ankle Power	0.2	0.2	0.2
Average Positive Lower Limb Power	0.2	0.2	0.2
Average Positive Biological Lower Limb Power	0.2	0.2	0.2
Soleus iEMG	0.067	0.121	0.2
Vastus Lateralis iEMG	0.085	0.196	0.145

Supplemental Table 2. Mean ± standard deviation of key parameters while walking with only biofeedback (BF), wearing shoes (baseline), with only ankle assistance (Assist), and with biofeedback-plus-assistance (BF+Assist). * indicates significant different from BF using paired two-tailed t-tests ($p \leq 0.05$). Values in blue font indicate reductions compared to BF. Values in red font indicate increases compared to BF.

Parameters	BF	Baseline	Assist	BF+Assist
Step length (m)	0.47 ± 0.09	0.41 ± 0.08*	0.43 ± 0.08*	0.47 ± 0.12
Peak Hip Extension Angle (°)	-15.9 ± 7.6	-12.8 ± 7.9	-14.1 ± 9.0	-18.4 ± 9.1*
Peak Knee Extension Angle (°)	7.9 ± 10.5	11.9 ± 4.7	9.0 ± 10.2	5.1 ± 11.5
Peak Ankle Plantarflexion Angle (°)	1.3 ± 3.7	-5.0 ± 7.8*	-0.2 ± 9.4	1.1 ± 6.7
Peak Hip Extension Moment (Nm/kg)	0.89 ± 0.26	0.57 ± 0.20*	0.74 ± 0.16*	0.83 ± 0.27
Peak Knee Extension Moment (Nm/kg)	0.76 ± 0.33	0.59 ± 0.19	0.61 ± 0.18	0.74 ± 0.27
Peak Ankle Plantarflexion Moment (Nm/kg)	1.27 ± 0.20	1.20 ± 0.22	1.41 ± 0.16*	1.36 ± 0.14*
Peak Biological Ankle Plantarflexion Moment (Nm/kg)	1.27 ± 0.20	1.20 ± 0.22	1.20 ± 0.18	1.15 ± 0.16*
Peak Support Moment (Nm/kg)	2.23 ± 0.55	1.69 ± 0.40*	2.04 ± 0.37	2.17 ± 0.49
Peak Biological Support Moment (Nm/kg)	2.23 ± 0.55	1.69 ± 0.40*	1.95 ± 0.38	2.09 ± 0.49
Average Positive Hip Power (W/kg)	0.60 ± 0.24	0.42 ± 0.16*	0.55 ± 0.22	0.57 ± 0.21
Average Positive Knee Power (W/kg)	0.30 ± 0.12	0.23 ± 0.13	0.28 ± 0.10	0.30 ± 0.15
Average Postivie Ankle Power (W/kg)	0.23 ± 0.09	0.25 ± 0.10	0.30 ± 0.08*	0.28 ± 0.09*
Average Positive Biological Ankle Power (W/kg)	0.23 ± 0.09	0.25 ± 0.10	0.27 ± 0.08	0.26 ± 0.08*
Average Positive Lower Limb Power (W/kg)	1.13 ± 0.39	0.90 ± 0.30*	1.12 ± 0.35	1.14 ± 0.36
Average Positive Biological Lower Limb Power (W/kg)	1.13 ± 0.39	0.90 ± 0.30*	1.09 ± 0.35	1.12 ± 0.36
Soleus iEMG (%baseline)	0.61 ± 0.15	0.58 ± 0.17	0.54 ± 0.14*	0.56 ± 0.16*
Vastus Lateralis iEMG (%baseline)	0.46 ± 0.13	0.31 ± 0.07*	0.33 ± 0.08*	0.42 ± 0.15
